# Supplementary figures and images for: PerfectlyAverage: A classical open‐source software method to determine the optimal averaging parameters in laser scanning fluorescence microscopy
Source: J Microsc. 2025 May 14;299(2):155–65. doi: 10.1111/jmi.13425 (PMC12265855; doi:10.1111/jmi.13425)

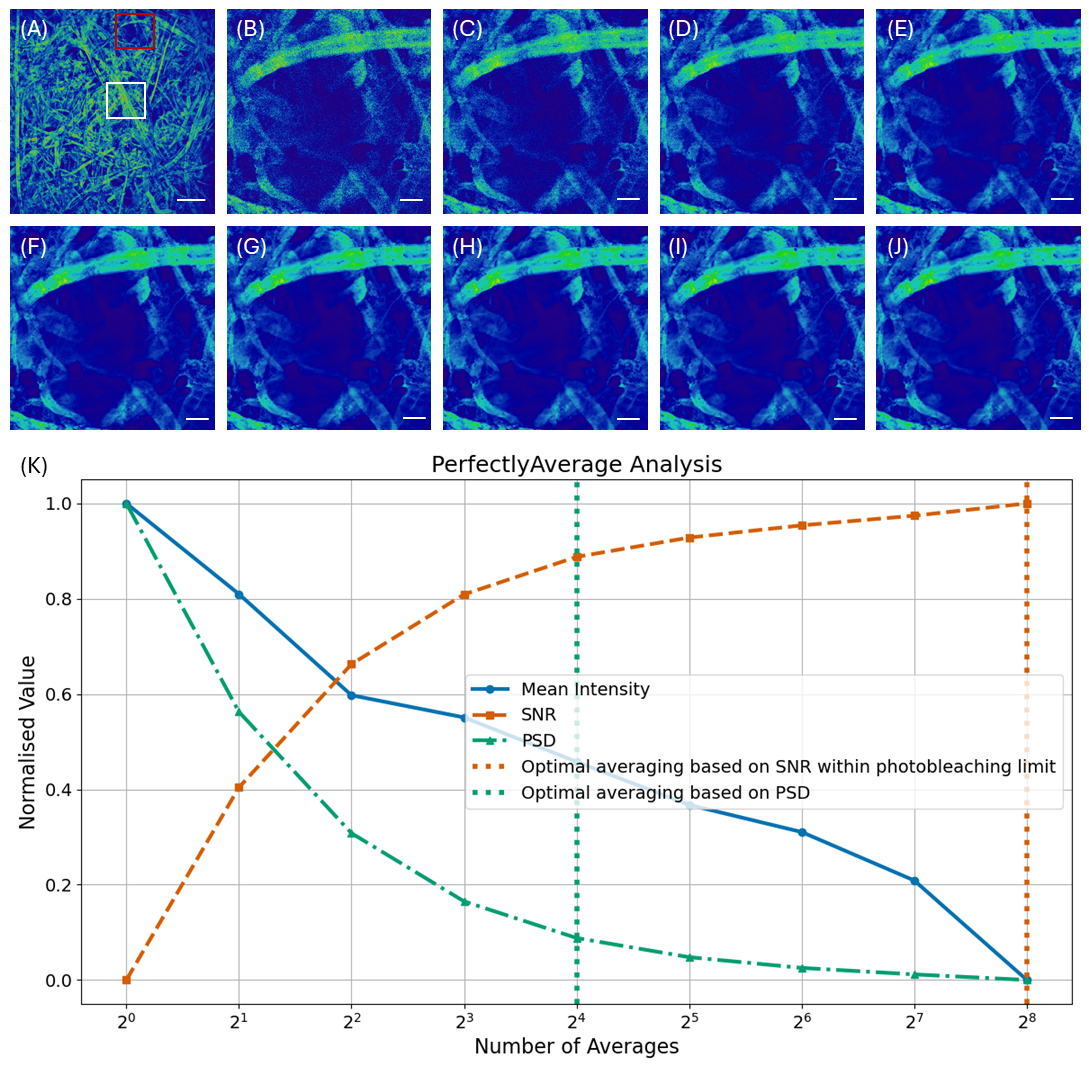

Supplement: Supplementary file 1 — Supporting Information. [file JMI-299-155-s002.png]

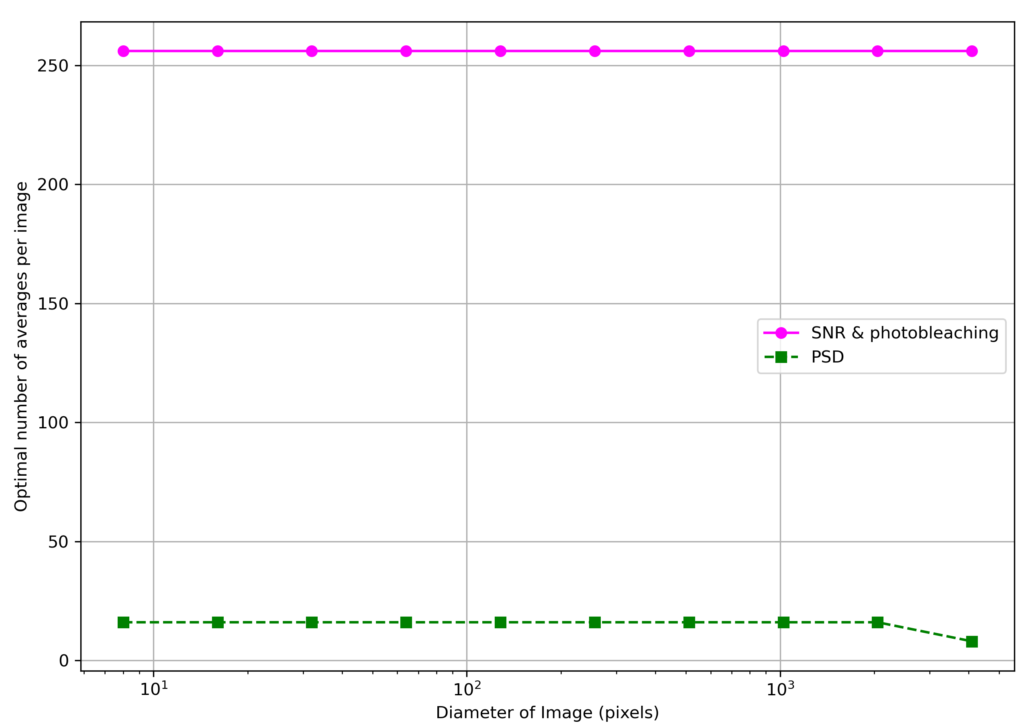

Supplement: Supplementary file 2 — Supporting Information. [file JMI-299-155-s001.png]
